# Targeting Oxidative Stress and Mitochondrial Dysfunction in the Treatment of Impaired Wound Healing: A Systematic Review

**DOI:** 10.3390/antiox7080098

**Published:** 2018-07-24

**Authors:** Mariola Cano Sanchez, Steve Lancel, Eric Boulanger, Remi Neviere

**Affiliations:** 1 Laboratory of Cardiovascular Physiology, Antilles University, P-97200 Fort de France, France; mariola.cano@uvic.cat; 2 Tissue Repair and Regeneration Laboratory (TR2Lab), University of Vic-UCC, Vic P-08500 Barcelone, Spain; 3 INSERM U1011-EGID, Institut Pasteur de Lille, Université Lille, CHU Lille, F-59000 Lille, France; slancel@univ-lille2.fr; 4 INSERM U995-LIRIC Inflammation Research International Centre, Université Lille, F-59000 Lille, France; eric.boulanger@univ-lille.fr; 5 Département de Gériatrie, CHU Lille, F-59000 Lille, France; 6 Département de Physiologie, CHU Lille, F-59000 Lille, France

**Keywords:** wound healing, reactive oxygen species, mitochondria, advanced glycation end products, diabetes, inflammation, antioxidants

## Abstract

Wound healing is a well-tuned biological process, which is achieved via consecutive and overlapping phases including hemostasis, inflammatory-related events, cell proliferation and tissue remodeling. Several factors can impair wound healing such as oxygenation defects, aging, and stress as well as deleterious health conditions such as infection, diabetes, alcohol overuse, smoking and impaired nutritional status. Growing evidence suggests that reactive oxygen species (ROS) are crucial regulators of several phases of healing processes. ROS are centrally involved in all wound healing processes as low concentrations of ROS generation are required for the fight against invading microorganisms and cell survival signaling. Excessive production of ROS or impaired ROS detoxification causes oxidative damage, which is the main cause of non-healing chronic wounds. In this context, experimental and clinical studies have revealed that antioxidant and anti-inflammatory strategies have proven beneficial in the non-healing state. Among available antioxidant strategies, treatments using mitochondrial-targeted antioxidants are of particular interest. Specifically, mitochondrial-targeted peptides such as elamipretide have the potential to mitigate mitochondrial dysfunction and aberrant inflammatory response through activation of nucleotide-binding oligomerization domain (NOD)-like family receptors, such as the pyrin domain containing 3 (NLRP3) inflammasome, nuclear factor-kappa B (NF-κB) signaling pathway inhibition, and nuclear factor (erythroid-derived 2)-like 2 (Nrf2).

## 1. Introduction

Wound healing is a well-tuned biological process, which is achieved via consecutive and overlapping phases including hemostasis, inflammatory-related events, cell proliferation and tissue remodeling [1,2]. Redox signaling and increased oxidative stress play a significant role in regulating normal wound healing by facilitating hemostasis, inflammation, angiogenesis, granulation tissue formation, wound closure, and development and maturation of the extracellular matrix [3]. ROS (reactive oxygen species) are centrally involved in all wound healing processes as low concentrations of ROS generation are required to fight against invading microorganisms and cell surviving signaling [3,4,5,6]. Excessive and uncontrolled oxidative stress contribute to sustaining and deregulating inflammation processes, which play a central role in the pathogenesis of chronic non-healing wounds [6,7]. In line, antioxidant and anti-inflammatory properties of several antioxidant strategies have proven beneficial to improve non-healing state [8,9,10]. This review will examine the role of redox signaling and oxidative stress in the etiology of impaired wound healing, with a particular focus on the development of treatment strategies based on mitochondrial-targeted antioxidants.

## 2. Normal Wound Healing and Redox Regulation

### 2.1. Wound Healing Phases

**Figure 1:**
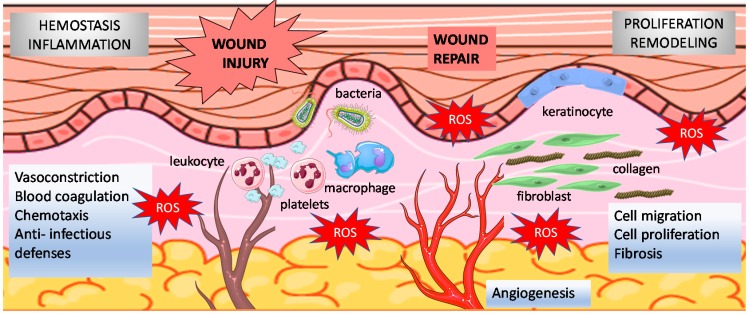
Normal wound healing phases. Relationships between wound healing and reactive oxygen species (ROS) are illustrated. In the hemostatic and inflammatory phase, large amounts of superoxide are generated from molecular oxygen mainly by NADPH oxidase expressed in immune cells. Redox signaling is also critical to modulate key events that occur during cell migration, proliferation, fibrosis and remodeling phases.

The complex molecular and cellular processes of wound healing occur in overlapping phases consisting of inflammation, formation of the granulation tissue including myofibroblast accumulation, extracellular matrix synthesis, angiogenesis, re-epithelialization, and tissue remodeling [1,2] (Figure 1).

Hemostasis typically begins immediately following injury in order to stop bleeding. Platelets are cells that are devoted to sealing off the injured blood vessel. First, platelets are plugged into the injured endothelium to form a clot, so-called primary hemostasis; this process is associated with platelet degranulation leading to the release in the extracellular milieu of cytoplasmic granules containing serotonin and thromboxane A2. These mediators induce vasoconstriction and thus limit blood loss through the disrupted vascular walls [11,12]. Ongoing accumulation of thrombin at the injured vascular site elicits recruitment of platelets to stimulate hemostatic plug growth. This phenomenon is associated with chemotaxis of many cell types such as macrophages, leukocytes, vascular smooth muscle cells and fibroblasts. In this pro-inflammatory milieu, platelets also release cytokines and growth factors, such as platelet-derived growth factor (PDGF), transforming growth factor (TGF)-β, fibroblast growth factor (FGF) and epidermal growth factor (EGF). Release of these growth factors presents potent signaling events for activation and proliferation of smooth muscle cells and fibroblasts concurring to blood vessel repair [13,14].

The inflammatory cells migrate to the injury site to fight infection and prepare the injured site for healing [15,16]. Migration of inflammatory cells at the site of injury participates to microorganism killing, preparing for successful healing [15,16]. In line, macrophages release TGF-β initiating a positive feedback loop as TGF-β further stimulates macrophages to secrete additional cytokines such as FGF, PDGF, tumor necrosis factor (TNF)-α and interleukin (IL)-1β [17]. Along with immune competent cell recruitment, mast cell activation and degranulation result in the release of prothombotic factors and antithrombotic/fibrinolytic components, histamine and other active amines, which cause increased endothelial permeability and edema allowing further mononuclear cells recruitment [17,18]. Overall, once vascular wall disruption is secured, chemotaxis promotes migration of inflammatory cells and initiate sequential infiltration of neutrophils, macrophages, and lymphocytes within the wound.

The proliferative phase generally follows and overlaps with the inflammatory phase. First, changes in macrophage phenotype allow for the transition of inflammation to proliferation and epithelial migration. As such, macrophages are responsible for initiation of cell apoptosis and clearance, including leukocytes, initiating the resolution of inflammatory processes [16,17,18]. Along with apoptotic cell clearance, transition of macrophage phenotype also initiates the recruitment of TGF-β released by platelets and macrophages stimulate production of collagen by fibroblasts. Secretions of growth factors (PDGF, FGF) and cytokines (TNF-α, IL-1β) stimulate in turn angiogenesis, fibroblast proliferation, collagen production and wound contraction [16,17,18]. Cross-talk between fibroblasts and keratinocytes is crucial to stimulate proliferation of keratinocytes which regenerates epithelium integrity. During the last few years, adipocytes from the dermis have been implicated in various physiological and pathological processes, among them hair follicle cycling, wound healing and cutaneous fibrosis. Specifically, adipocyte precursor cells and/or mature adipocytes have been implicated in several regenerative and pathological processes in the skin including hair follicle regeneration and fibroblast regeneration after injury.

During the proliferative stage of wound healing, release of cytokines and growth factors, such as TGF-β, stimulates the migration of keratinocytes and fibroblasts toward the wound area to begin the re-epithelialization and tissue rebuilding process. The proliferative phase involves the conversion of fibroblasts into myofibroblasts that secrete extracelluar matrix ECM proteins, which are required for closure of the wound [19,20]. Myofibroblasts also exhibit contractile properties, due to the expression of α- smooth muscle actin in microfilament bundles or stress fibers, playing a major role in contraction and in maturation of the granulation tissue [19,20]. Depending on experimental or clinical situation, myofibroblasts can also express other smooth muscle related contractile proteins, such as desmin or smooth muscle-myosin heavy chain.

Remodeling and maturation phases allow the termination of wound repair. During these processes, rates of collagen synthesis collagen breakdown equilibrate. During remodeling processes, proteolytic enzymes, essentially matrix metalloproteinases (MMPs), and their inhibitors (tissue inhibitor of metalloproteinases, TIMPs) play a major role. Deposit disorganized collagen in the wound, it is cross-linked and aligned along tissue tension lines. More fibroblasts are gathered in the new connective tissue, so-called granulation tissue, which lay down collagen, eventually resulting in the formation of a scar [17,18,19]. At this point, synthesis of ECM is considerably reduced. In addition, synthesized components are modified as the matrix is remodeled. Progressively, type I collagen, the main structural component of the dermis, replaces type III collagen which is the major collagenous component of granulation tissue. Finally, elastin which is absent in the granulation tissue and normally contributes to skin elasticity also reappears. During the resolution phase of healing, generally, when the tissue integrity has been sufficiently restored to be mechanically coherent, cellularity is drastically reduced by apoptosis of both myofibroblasts and vascular cells [17,18,19,20].

### 2.2. Redox Regulation of Normal Wound Healing

Oxygen is required to disinfect wounds and provide adequate fuel for healing. In addition, oxygen (O_2_)-dependent redox signaling is crucial for wound repair [3]. Physiologically, hydrogen peroxide (H_2_O_2_) and superoxide serve as intracellular messengers stimulating key phases of wound healing including cell recruitment, production of cytokines and angiogenesis [3,4,5,6] (Figure 1). Of note, H_2_O_2_, a reactive species produced by dismutation of superoxide, acts as the principal secondary messenger in wound healing and is present at low concentrations (100–250 μM) in normal wounds [21].

Current understanding of the role of homeostatic levels ROS and redox signaling in wound healing has been well established [3,4,5,6,7]. Physiological levels of ROS are important to reduce local blood flow via vasoconstriction and thrombus formation. Early onset ROS peak levels are related to initial platelet aggregation, which contribute to platelet and inflammatory cell recruitment to the wound by stimulating chemotaxis and adhesion molecule expression [22]. Second, release of ROS within tissue stimulates diapedesis of adherent leukocytes across the vascular wall in order to induce microorganism killing at the injured site. High levels of superoxide and H_2_O_2_ are thus generated by neutrophils and macrophages via NADPH oxidase [22]. This oxidative burst serves as the primary mechanism of bacterial killing and prevention of wound infection, and is accompanied by a temporary down-regulation of some ROS scavenging enzymes [23]. ROS also provide further signals supporting wound repair as evidenced by their stimulating effects on tumor necrosis factor-α (TNF-α) and platelet-derived growth factor (PDGF) release. Other immune competent cells, including monocytes and macrophages, migrate towards the wound site to help attack invading pathogens [22,23]. Redox signaling is also critical for the proliferation phase. ROS promote fibroblast proliferation and migration, and mediate TGF-β1 signaling, which results in migration, collagen and fibronectin production, and basic fibroblast growth factor (FGF) expression [22,24]. ROS also stimulates angiogenesis, endothelial cell division and migration for blood vessel reformation via VEGF expression. ROS facilitate wound edge and stimulated both proliferation and migration of fibroblasts leading to ECM formation. At the same time, keratinocyte proliferation and migration are promoted, facilitating re-epithelialization [22].

Generation of ROS during the hemostatic phase of wound healing is related to NADPH oxidases (NOX) located in vascular cells, which are activated by tissue factor expression secreted by platelets [22,24,25]. During inflammation, ROS production by NOX enzymes plays a central role in microorganism killing by neutrophil and macrophage oxidative burst [22,23,24]. Whereas the NOX2 isoform is responsible for the large amounts of ROS produced during the respiratory burst, NOX4 isoform has been implicated in phagocyte recruitment [22,25]. Production of ROS by NOX enzymes is also involved in wound angiogenesis and in the regulation of re-epithelialization processes favoring effective wound closure [22]. Overall, crucial functions of inflammatory cells, vascular endothelial cells, fibroblasts and epithelial cells in wound healing are related to their potent ability to produce ROS via NOX activation [22].

Key events of wound re-epithelialization are dependent on adequate migration, proliferation and differentiation of keratinocytes. Eventually, wound contraction, which guarantees rapid wound closure, is under redox control as ROS produced by NOX4 have shown to be a prerequisite for TGF1-induced myofibroblast differentiation, extracellular matrix production. After wound closure, controlled cell removal and ECM remodeling are dependent upon NOX-mediated ROS production, which is likely to play a direct role during scar maturation [22,25]. ROS generated in wounds is tightly regulated by ROS scavenging enzymes, such as superoxide dismutases (Cu/ZnSOD, MnSOD, SOD3), peroxidases (catalase, phospholipid hydroperoxide glutathione peroxidase) and peroxiredoxins, as well as small molecule antioxidants, such as vitamin E and glutathione [8,9,10]. Many of the enzymes are up-regulated in healing wounds, while levels of small molecule antioxidants decrease as depleted by ROS [8,9,10]. Overall, a balance of ROS generation and scavenging is required for efficient and timely wound healing.

## 3. Definition of Chronic Wound

Several pathologic conditions result in an incomplete and prolonged healing process [2]. Pathogenesis of chronic wounds is difficult to study due to many factors including complexity of the wound repair process and heterogeneity of chronic wounds. Local ischemia and reperfusion injury, type 2 diabetes, chronic inflammation, aging and senescence are the most important factors that may elicit chronic wound states [2,3,4].

Among these factors, the chronic state of inflammation is present in the majority of cases [16]. Inflammation is associated with persistent macrophages which limit and delay proliferation. Chronic inflammation also induces cell senescence, which is now considered as a critical pathophysiological process in the development of chronic wounds [16,26]. A chronic wound environment is characterized by elevated levels of proteases such as matrix metalloproteinase (MMPs), reduced levels of protease inhibitors such as tissue inhibitors of mMMP (TIMPs), and an abundance of inflammatory cells releasing excessive amounts of proinflammatory cytokines, proteolytic enzymes, and ROS [27]. A combination of these factors elicits accelerated degradation of extracellular matrix and growth factors, deregulation of the inflammatory response, inhibition of cellular proliferation, inadequate vascularization, and accumulation of necrotic tissue due to ischemia. In turn, these effects encourage bacterial colonization and can perpetuate the inflammatory response, inhibiting wound repair.

## 4. Oxidative Stress in Chronic Wound

As discussed above, a delicate balance between the positive role of ROS and their deleterious effects is important for proper wound healing. Whereas production of ROS is essential to initiate wound repair, excessive amount of ROS generation is deleterious in wound healing. Ongoing oxidative stress, associated with lipid peroxidation, protein modification and DNA damage has been shown to impair wound healing processes via increased cell apoptosis and senescence [3,4,5,6,7]. In physiological conditions, low levels of ROS production by NOX activation in neutrophils and macrophages are responsible for respiratory bursts during phagocytosis of the inflammatory phase [22,23,24]. In contrast, as chronic inflammation develops in pathological conditions, NOX activation is exacerbated, which may lead to excessive production of ROS production, further accelerating inflammation and oxidative stress cellular damage. Clinical studies suggest that non-healing wounds are maintained in highly oxidizing environment, which lead to impaired wound repair. Clinical conditions such as tissue hypoxia and hyperglycemia are typically associated with highly oxidizing environments.

### 4.1. Hypoxic Wound

Whereas generation of ROS during the normal wound healing is related to NOX activation [22,23,24], the presence of hypoxia stimulates oxidant production by the electron transport chain (ETC) of the mitochondria mainly via complexes I and III [28]. This observation is paradoxical, in the sense that superoxide is a product of the one-electron reduction of O_2_, which is reduced in hypoxia. ETC-derived ROS are transferred across the inter-membrane space to reach the cytosol where they act as second messengers. During hypoxia, mitochondria augment the release of ROS in the cytosol, which appears counter intuitive as O_2_ tension is reduced in the mitochondrial compartment [28,29]. Hypoxia-induced mitochondrial ROS release has been shown to activate cell protection signaling through transcriptional and post-translational mechanisms [28,29].

In line, low oxygen levels leading to mitochondrial ROS production activate prolyl-4-hydroxylases. Prolyl-4-hydroxyases can induce hypoxia-inducible factor 1 (HIF-1) activation, which is involved in regeneration of lost or damaged tissue in mammals [29,30]. In the microenvironment of early wounds, ischemia due to vascular disruption and high O_2_ consumption by immune competent cells can favor O_2_ depletion and hypoxia [2,6]. Moreover, pathological conditions, such as diabetes, impair microvascular blood flow, thus aggravating tissue oxygenation [2], whereas temporary hypoxia after injury can be beneficial for wound healing, prolonged or chronic hypoxia delays wound healing. Impaired wound repair in hypoxic tissue has been related to the combination of mechanisms that increase ROS production and reduce antioxidant defenses [6].

### 4.2. Diabetic Chronic Wound

ROS production by several ROS-generating enzymes is elevated in diabetic wounds [10]. Expression and activity of NOX, the major source of ROS in many cell types, are increased in response to hyperglycemia through activation of the receptor for advanced glycation end products (RAGE) [31]. NOX activity is also increased downstream of hyperglycemia-induced protein kinase C (PKC) activation in smooth muscle and endothelial cells [32]. Similarly, hyperglycemia-induced angiotensin II type 1 receptor AT1 activation increases expression of p47phox and enhances ROS production by NADPH oxidase [33]. AT1 is expressed by several cell types in the wound, including myofibroblasts and keratinocytes [34]. Expression and activity of H_2_O_2_-producing enzyme xanthine oxidase (XO) is also increased in diabetic mouse wounds and in response to high glucose levels [35].

One of the most important sources of ROS in diabetes is the mitochondrial electron transport chain [32]. In line, hyperglycemia increases superoxide production by increasing the amount of pyruvate oxidation in the TCA cycle and consequently the availability of electron donors NADH and FADH2 [32,36]. Increased electron flux then increases the proton gradient across the inner mitochondrial membrane, which at a critical threshold disrupts electron transport through complex III [36]. Electron transport is then largely mediated by coenzyme Q, which transfers only one electron to oxygen, producing excess superoxide [36]. Excessive mitochondrial superoxide production further impacts ROS levels by altering the flux through several intracellular pathways. For example, ROS leads to GAPDH inhibition by poly (ADP-ribose) modification, which increases levels of glycolysis intermediates upstream of GAPDH [32,36]. This provides increased substrate levels for the polyol, protein kinase C, and hexosamine pathways [32,36]. Activation and interaction of these pathways ultimately alters gene expression, depletes antioxidant resources, and favors the production of further ROS and advanced glycation end products. In addition, multiple lines of evidence have emerged showing that intracellular sites of ROS production are functionally connected. So-called ROS-induced ROS release cross talk represents a common mechanism for ROS amplification and regional ROS generation [37]. A large number of mitochondrial pores (mPTP, inner membrane anion channel (IMAC), voltage dependent anion channels VDAC) has been identified as facilitating superoxide escape to the cytosol [37,38].

Hyperglycemia, mitochondrial ROS generation, and oxidative stress are involved in the pathogenesis of several diabetic complications. Deleterious effects of ROS on cellular homeostasis are also related to the reduction in antioxidant defenses, which intensifies the redox imbalance. Analysis of blood collected from diabetes patients showed reduced SOD, CAT, and glutathione peroxidase activity, and an overall decrease in antioxidant status [39]. Of note, signaling through the transcription factor nuclear factor erythroid 2-related factor 2 (Nrf2), a master regulator of antioxidant gene expression, is impaired in diabetes [40]. Expression and nuclear translocation of Nrf2 are decreased in diabetic dermal fibroblasts. In response to oxidative stress, Nrf2 activity decrease was associated with reductions in expression of CAT, NADPH dehydrogenase quinone 1 (NOQ1), glutathione reductase, and glutathione S-transferase [41]. In fibroblasts exposed to high glucose concentrations, Nrf2 is retained in the cytoplasm by its regulator Keap1, and transcription of MnSOD and NOQ1 is reduced [42]. Activation of ATF-3 and NF-κB is involved in antioxidant enzyme regulation is also altered in response to foot ulceration in diabetic patients [43].

## 5. Glycoxidation and AGE Formation in the Diabetic Chronic Wound

AGEs are produced by the Maillard reaction. A reducing sugar, such as glucose, reacts non-enzymatically with the amino group of proteins to produce a Schiff base that rearranges into Amadori compounds [5]. These Amadori adducts then very slowly undergo irreversible dehydration and condensation reactions, eventually producing AGEs, which are yellowish-brown materials with particular fluorescence.

**Figure 2:**
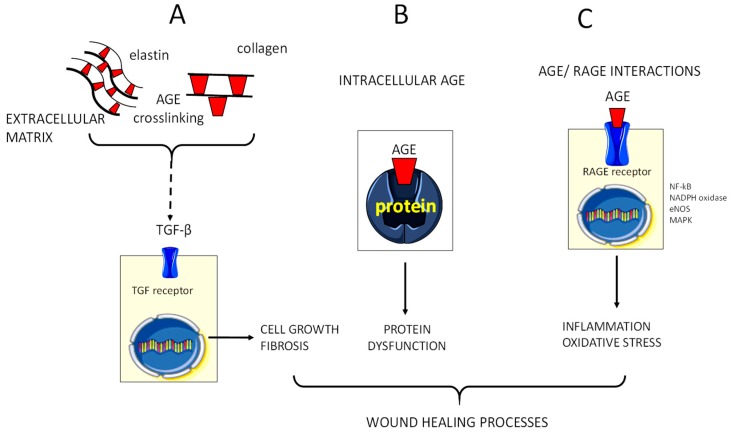
Advanced glycation end products AGE (advanced glycation end products) modulate wound healing. Effects of AGE accumulation in the extracellular (**A**) and intracellular environment (**B**), and interactions with the receptor RAGE (**C**) are illustrated. In extracellular matrix, AGE form on different molecules as collagen, laminin and elastin, increasing matrix stiffness. AGE products upregulate transforming growth factor (TGF)-β that increases the production of extracellular matrix components by binding to TGF β receptor. AGE interact with RAGE on the cell surface leading to transduction of signaling cascade, which activates the ROS generating NADPH oxidase and mitogen-activated protein kinases (MAPK). A main step in AGE/RAGE signaling is activation of NF-κB and its translocation to the nucleus, where it enhances transcription of target genes involved in the inflammatory response. AGE may decrease nitric oxide (NO) availability by reducing endothelial nitric oxide synthase (eNOS) activity and by inactivating NOS elicits ROS production.

Advanced glycation end products (AGEs) are a heterogeneous group of compounds produced by the non-enzymatic Maillard reaction. In this reaction, a reducing sugar reacts with the amino group of proteins to form a Schiff base that form Amadori structures. These adducts then slowly transform to eventually produce AGE [44]. Formation of AGE is a physiological process that is part of normal metabolism during lifetime and accumulates slowly in human tissues during ageing [45,46]. However, more rapid and intense accumulation occurs in association with consistent hyperglycemia and enhanced oxidative or carbonyl stress [44]. In addition, glycoxidation, a combinational effect of oxidation and glycation, causes formation of dicarbonyls methyleglyoxal and glyoxal which further fuels AGE formation [44]. Proposed mechanisms of AGE-induced diabetic complications include accumulation of AGE in the extracellular matrix causing aberrant crosslinking and vessel rigidity increase. AGE also bind to AGE-receptors leading to the activation of key cell signaling pathways such as NADPH oxidase and NF-κB activation. Eventually, intracellular formation may impair biomolecule and protein functions including nitric oxide quenching and growth factor effects [45,46] (Figure 2). In line, complications of diabetes such as micro and macrovascular complications, neuropathy and impaired wound healing have attributed to AGE formation and glycoxidation within tissues [45,46].

An increasing body of in vitro and in vivo experimental diabetic models as well as anti-AGE agents suggest that AGE contribute to the impaired wound healing [47,48]. First, AGE formation may impair micro and macrovascular function leading to vascular flow deficit and poor tissue oxygenation [49]. In addition, increased AGE accumulation, along with expression of their receptors, primarily the RAGE, are associated with formation of atheromatous lesions, impairing blood flow as well as ischemia-induced neovascularization and formation of collateral circulation [50]. Second, either AGE accumulation in diabetic tissues or activation of RAGE have been implicated in the decreased transendothelial migration of neutrophils, which ultimately concurs to wound hypocellularity and delayed inflammatory response [48,51]. In addition, binding of AGE to RAGE induced ROS production from inflammatory and endothelial cells via NADPH activation, which promotes further cellular activation and proinflammatory cytokine expression [52]. Wound AGE accumulation can sustain inflammatory response eliciting ROS production, which further increases AGE formation creating a vicious cycle. Third, a large body of evidence demonstrates that activation of the AGE/RAGE axis alters locomotion, invasiveness, phenotype, behavior, and survival of the cells as well as cell membrane interactions with the extracellular matrix [48]. AGE/RAGE activation in the diabetic skin is associated with extracellular matrix glycation leading to cell cycle arrest of cultured dermal fibroblasts [52]. AGE also activates apoptosis of dermal fibroblasts that would deprive cell proliferative processes necessary for proper behavior. Eventually, accumulation of AGE is associated with derangement in wound contraction and remodeling in diabetic *db/db* mice [53]. In this diabetic mouse model, lowering circulating and tissue accumulating AGE by low AGE diet has been shown to improve wound repair [54]. In addition, delayed epidermal regeneration, thin granulation tissue formation, and impaired neovascularization that led to a significant delay in wound contraction and wound closure were almost reversed by blocking RAGE [55].

In humans, activation of the AGE/RAGE axis has been associated with chronic wound states featured by decreased epithelialization and angiogenesis, deregulation of inflammation, impaired granulation tissue deposition and collagen organization, as well as increased contraction and delayed wound closure [48,50]. Studies in skin of diabetic patients suggest that accumulation of AGEs in fibroblasts is associated with oxidative damage and can alter dermis characteristics of diabetic patients including reduced dermis thickness, disorganization of collagen fibrils, and infiltration of inflammatory cells [52]. In these experiments, RAGE-blocking antibodies prevented glycosylated matrix induced cell cycle arrest and apoptosis of cultured dermal fibroblasts [52]. Interestingly, use of topical soluble RAGE treated wounds also shown increased granulation tissue area and micro-vascular density [55]. Overall, growing evidence suggests that AGE accumulation is involved in dermal wound healing dysfunction and that its prevention could represent a valuable therapeutic option to improve wound healing in diabetic patients.

## 6. Redox Balance Modules Inflammation in the Healing Wound

Inflammation is a key step to prepare the onset of wound repair [1,7,15,16]. However, resolution of inflammation delay can result in a chronic and deregulated response creating further tissue damage [23]. As previously mentioned, increased and prolonged levels of ROS production in the wound result in chronic inflammation. Interestingly, restoring the redox balance has been shown to improve inflammatory skin conditions.

Excessive presence of ROS in the skin promotes activation of a variety of transcription factors including nuclear factor kappa B (NF-kB), activator protein 1 (AP-1), nuclear factor erythroid-derived 2-like 2 (Nrf2), and mitogen-activated protein kinase (MAPK) pathways [3,4,5,6]. Nrf2 is the master regulator of antioxidant gene expression that regulates the transcription of cytoprotective genes by binding to the antioxidant response element (ARE) to activate the transcription of its target genes [39,40]. The main function of Nrf2 during wound healing is protection against the excessive accumulation of endogenous ROS. Growing evidence suggest that overexpression of Nrf2 can limit cellular damage induced by oxidative stress. In addition, Nrf2 expression is essential for the regulation of re-epithelialization processes [39,40,41,42]. In the opposite direction, activation of NF-kB and AP-1 has been shown to increase matrix metalloproteinase (MMP) activity in dermal fibroblasts resulting in extracellular matrix (ECM) protein degradation and features of premature aging of the skin. Hence, Nfr2 and NF-κB have reciprocal role in wound healing. On the one hand, Nrf2 controls inflammation and ROS production. On the other hand, Nf-κB can activate the innate immune response, regulate proliferation and migration of many cell types, activate matrix metalloproteinases, and alter cytokines and growth factor cell functions [56]. Interestingly, Nrf2 expression in chronic wounds has been related to major down-regulation of in the diabetic mice as well as increases in protein oxidation in the skin of diabetic patients. Overall, the existing literature supports the contention that Nrf2 activators could improve wound healing in diabetic conditions [40,41,42].

**Figure 3:**
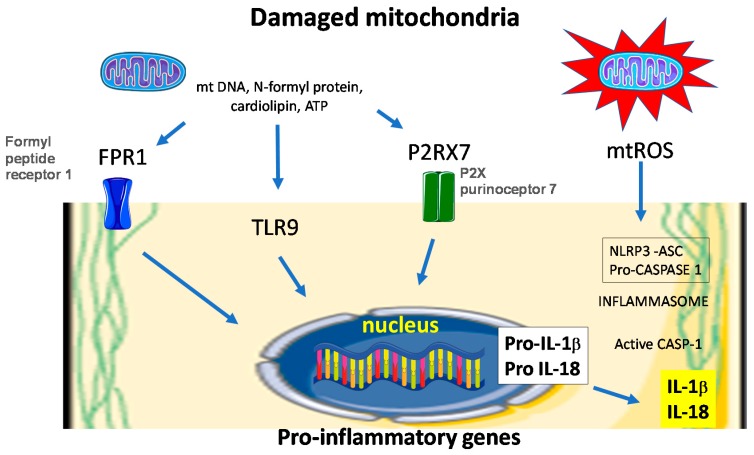
Mitochondria dysfunction elicits inflammasome activation. Injured mitochondria release molecular pattern that are recognized by cell membrane receptors and cytosolic toll-like receptor (TLR) 9. NLRP3 inflammasomes are activated by a myriad of stimuli that include danger-associated molecular patterns (DAMPs). Once activated, NLRP3 forms a multimeric protein complex with associated speck- like protein containing a caspase activation and recruitment domain (CARD; ASC) and caspase-1 (CASP1) termed the inflammasome. Caspase-1 is activated in the inflammasome complex, which cleaves pro-IL-1β (pro-interleukin-1β) and pro-IL-18 into their bioactive mature forms. Mitochondrial DNA (mtDNA), N-formyl proteins, ATP and mitochondrial reactive oxygen species (mtROS), have all been shown to promote NLRP3 inflammasome activation either directly or via specific receptor such as formyl peptide receptor 1 (FPR1) and P2X purinoceptor 7 (P2RX7). TLR9 preferentially binds DNA motifs present in mitochondria and triggers signaling cascades that lead to a pro-inflammatory cytokine response.

Deregulation of the inflammatory phase of wound healing and persistence of the pro-inflammatory macrophages in the diabetic wounds has been related to sustained NLRP3 inflammasome activity [57]. NLRP3 inflammasomes are multiprotein complexes with an inherent ability to elicit innate immune responses by sensing pathogen-associated molecular patterns (PAMP) and damage-associated molecular signals (DAMP) through NOD-like receptors (NLR) family pyrin domain containing 3 (NLRP3) binding. Upon activation by PAMPs or DAMPs, NLRP3 interacts with the adapter protein apoptosis associated speck-like protein (ASC). Then, the caspase recruitment domain (CARD) of ASC binds to the CARD domain on procaspase-1, forming the NLRP3 inflammasome. The assembled complexes act as proteolytic cleavers which activate the precursor of interleukin (IL)-1β and IL-18, which are involved in a series of immune and inflammatory processes (Figure 3). Inflammasomes are associated with an antimicrobial response as well as numerous autoimmune, autoinflammatory, metabolic, and infectious diseases.

In vitro and preclinical studies suggest that the NLRP-3 inflammasome is a key regulatory pathway in wound healing [58,59]. In vitro and experimental studies indicated that the NLRP3 inflammasome may be activated via a ROS-mediated pathway in the diabetic wound environment [60]. In these studies, downstream targets of NLRP3, namely IL-1β and IL-18 contribute to the sustained inflammatory response. Strategies aimed to inhibit NLRP3 inflammasome in vivo, such as topical application of pharmacological inhibitors, NLRP-3 bone marrow transfer and deletion of caspase-1 in *db/db* mice, have been shown to beneficially alter wound repair. In these studies, modulation of NLRP3 inflammasome was associated with an improved healing response and down-regulation of a pro-inflammatory phenotype of macrophages [58,59,60].

Growing evidence suggests that ROS originating from mitochondria are centrally involved in NLRP3/NALP3 inflammasome activation, which is required to direct the proteolytic maturation of inflammatory cytokines such as IL-1β [61]. One may thus hypothesize that excessive wound inflammation in diabetic patients may be improved by mitochondria-targeted antioxidants in compromised wound healing. This contention is supported by recent studies showing that antioxidant SkQ1 may improve dermal wound healing in genetically diabetic mice [62]. In these experiments, prevention of excessive mitochondrial ROS production by SkQ1 improved resolution of the inflammatory phase, simultaneously decreasing content of neutrophils and increasing content of macrophages [62]. It seems reasonable to suggest that beneficial effects of SkQ1 on the inflamed wound could be attributed, at least in part, to the reduction of NLRP3 inflammasome activity, as these mitochondria-targeted ROS scavengers have been shown to decrease IL-1β and IL-18 production [63,64].

## 7. Targeting Oxidative Stress in the Treatment of Chronic Wound

In diabetic patients, standard wound care practices include surgical debridement, antibiotic treatment, moisture dressing, and pressure off-loading [1,2]. Recent advances have focused on specific defects in the diabetic wound environment, including topical application of growth factors, introduction of bone marrow-derived endothelial and epithelial cells, and collagen-based tissue-engineered grafts. As a different concept, strict control of ROS levels through antioxidants and antioxidative enzyme systems may reduced oxidative stress-induced cellular damage [65]. In line, studies using gene-modified animals and pathological models have shown beneficial effects of antioxidative enzyme upregulation in normal wound healing. For example, deficiency of SOD1, heme oxygenase (HO)-1 can delays wound healing processes in mice [66,67]. Reducing excessive ROS by the means of growth factor treatment, antioxidant N-acetyl cysteine or dietary antioxidants generation has been shown beneficial in experimental models of chronic wound. For example, decreasing activity of XO by topical application of siRNA targeting its precursor, xanthine dehydrogenase, significantly improves healing in db/db diabetic mice [68]. Similarly, genetic deletion of the H_2_O_2_-generating enzyme p66Shc elicited reduction of nitrosative oxidative stress and improved healing rate in diabetic mice [69]. Increasing antioxidant capacity via in vivo MnSOD transfer expression has also proven to be effective in diabetic mice [70]. Activation of Nrf2-mediated antioxidant defenses has been clearly associated in the recent literature with protection against diabetic wound healing in mice [39,40,41]. In human cells, inducers of Nrf2 have been suggested as a promising pharmacological strategy for skin photoprotection [71].

The next step in the evaluation of antioxidant-based therapy of chronic wound in humans may be clinical use of strategies targeting mitochondria ROS production. Mitochondria-targeted antioxidants have been initially designed to deliver treatments to mitochondria in cardiovascular and neurodegenerative diseases in order to reduce ROS and mitochondrial dysfunction [72,73]. Mitochondria-targeted antioxidants include triphenylphosphonium lipophilic cation-based molecules such as MitoQ Mito-αlipoic acid and 10-(6′-plastoquinonyl) SkQ1, small-cell permeable tetra peptide molecules such as elamipretide choline esters of glutathione and N-acetylL-cysteine [72,73]. As mentioned above, 10-(6′-plastoquinonyl) SkQ1 antioxidant may improve dermal wound healing via better resolution of inflammation in genetically diabetic mice [62]. Mitochondrial-targeted antioxidant elamipretide can bind to cardiolipin, reduce ROS production and stabilize mitochondrial function. Human studies have already shown that elamipretide improves heart failure and mitochondrial myopathy [74,75]. It is likely that elamipretide would also improve chronic wounds, thanks to effects of this compound on mitochondrial ROS production, NLRP3 inflammasome activity as well as NF-κB and Nrf2-ARE signaling pathways.

## 8. Conclusions

When combined with the successful pre-clinical models described above, promising clinical data suggest the value of redox-based therapeutics for wound healing in diabetic and hypoxic environments. There must be further development of current antioxidant treatment strategies and evaluation of new targets to address imbalances in redox signaling in these clinical situations. Rationale for the use of mitochondrial-targeted antioxidant strategies came from their beneficial effects on oxidative damage, exaggerated inflammatory response, apoptosis and senescence, all of which are implicated in chronic wounds.
